# Consensus-Phenotype Integration of Transcriptomic and Metabolomic Data Implies a Role for Metabolism in the Chemosensitivity of Tumour Cells

**DOI:** 10.1371/journal.pcbi.1001113

**Published:** 2011-03-31

**Authors:** Rachel Cavill, Atanas Kamburov, James K. Ellis, Toby J. Athersuch, Marcus S. C. Blagrove, Ralf Herwig, Timothy M. D. Ebbels, Hector C. Keun

**Affiliations:** 1 Biomolecular Medicine, Department of Surgery and Cancer, Faculty of Medicine, Imperial College London, London, United Kingdom; 2 Max Planck Institute for Molecular Genetics, Berlin, Germany; 3 MRC-HPA Centre for Environment and Health, Department of Epidemiology and Biostatistics, School of Public Health, Faculty of Medicine, Imperial College London, London, United Kingdom; 4 Department of Zoology, Oxford University, Oxford, United Kingdom; Lilly Singapore Centre for Drug Discovery, Singapore

## Abstract

Using transcriptomic and metabolomic measurements from the NCI60 cell line panel, together with a novel approach to integration of molecular profile data, we show that the biochemical pathways associated with tumour cell chemosensitivity to platinum-based drugs are highly coincident, i.e. they describe a consensus phenotype. Direct integration of metabolome and transcriptome data at the point of pathway analysis improved the detection of consensus pathways by 76%, and revealed associations between platinum sensitivity and several metabolic pathways that were not visible from transcriptome analysis alone. These pathways included the TCA cycle and pyruvate metabolism, lipoprotein uptake and nucleotide synthesis by both salvage and de novo pathways. Extending the approach across a wide panel of chemotherapeutics, we confirmed the specificity of the metabolic pathway associations to platinum sensitivity. We conclude that metabolic phenotyping could play a role in predicting response to platinum chemotherapy and that consensus-phenotype integration of molecular profiling data is a powerful and versatile tool for both biomarker discovery and for exploring the complex relationships between biological pathways and drug response.

## Author Summary

Resistance to chemotherapy drugs in cancer sufferers is very common. Using a panel of 59 cell lines obtained from different types of cancer we study the links between the genes and metabolites measured in these cells and the resistance the cells show to common cancer drugs containing platinum. In order to combine the information given by the genes and metabolites we introduce a new pathway-based approach, which allows us to explore synergy between the different types of data. We then extend the procedure to look at a wider panel of drugs and show that the pathways we found were associated with platinum are not just the pathways which are frequently selected for a large number of drugs. Given the increasing use of multiple sets of measurements (genes, metabolites, proteins etc.) in biological studies, we demonstrate a powerful, yet straightforward method for dealing with the resulting large datasets and integrating their knowledge. We believe that this work could contribute to developing a personalised medicine approach to treating tumours, where the genetic and metabolic changes in the tumour are measured and then used for prediction of the optimal treatment regime.

## Introduction

In the quest to understand complex biological systems at multiple levels of biological organization, the need arises to combine knowledge from experiments of different types to create a full picture of a system's behavior. Modern molecular profiling (“omics”) methods, such as transcriptomics, proteomics and metabolomics allow one to build up a global picture of system characteristics, and to search for interactions and coordinated behavior between the different levels. While each level can be studied separately, greater statistical and explanatory power can be gained by integrating this knowledge into a single coherent model of the system. This is currently one of the greatest challenges in systems biology.

Inter-omic data integration can be performed at different levels [1], the simplest of which is conceptual integration. At this level, each omics data set is analysed separately and a coherent biological rationale is constructed which explains phenomena observed in the separate molecular profiles. For example, changes in levels of both enzyme transcripts and metabolites from the same pathway could be explained by the hypothesis of differential regulation of that pathway. However this subjective approach can lead to plausible biological explanations that arise through spurious statistical associations and conversely some potentially novel mechanisms may be overlooked. The statistical level of integration is more objective. In this approach, links between data sets are made using rigorous statistical procedures such as correlation, regression or more sophisticated techniques. To date, much inter-omic data integration has been performed at the conceptual level [2], [3], [4] while various methods have been proposed and demonstrated for statistical integration [5], [6], [7], [8], [9].

Many researchers have found that interpretation of omics data at the level of individual molecular entities can be difficult and have opted for an analysis at the pathway or functional level [10]. This is mainly because particular changes in biochemical pathways, associated with phenotypic conditions such as disease can often arise from a range of different alterations in a pathway. A common method for performing pathway-level analysis on single omic data is over-representation (OR) analysis [11], [12], in which a set of molecular elements (e.g. genes) that are differentially expressed or correlated with the phenotype of interest are first selected. The set is then compared against molecular sets defined *a priori* (e.g. genes in established pathways) to identify those sets that show greater overlap with the phenotype-associated genes than would be expected by chance. The final list of significantly over-represented or ‘enriched’ sets/pathways is used to aid biological interpretation of the data. As well as performing OR with genes, Metabolite Set Enrichment Analysis (MSEA) [13] and other metabolite over-representation techniques [14] have also been developed. In this work we contrast the application of the OR analysis approach to transcript and metabolite data individually to the alternative of considering them simultaneously, using established pathways to guide an integrated analysis of the two data sets.

In addition to the inter-omic integration of metabolomic and transcriptomic data, our approach involves a further type of data integration that we call consensus-phenotype integration. In this approach, several examples of the same phenotype, achieved in different ways, are used within the experimental design. For example, one may study a particular mechanism of toxicity via the use of different chemical treatments that have a similar mode of action. One can thus identify features that are central to the phenotype in question across different types of “omics” data, as opposed to features that are specific to a single instance of the phenotype being studied.

In this work, we aim to elucidate mechanisms of drug sensitivity through the use of inter-omic statistical data integration using drug sensitivity, transcriptomic and metabolomic data from the NCI60 cell line panel [15]. The NCI60 is a panel of tumor derived cell lines corresponding to diverse tissue types, which has been subject to extensive molecular phenotypic and pharmacological characterization. We used baseline (untreated) metabolic and transcriptional profiles readily available for 58 lines as well as growth inhibition data from an array of 118 drugs [15], [16]. We correlate growth inhibition to the molecular profiles to identify pathways related to drug sensitivity. We first focus on platinum sensitivity as it is a well-defined phenotype, linked to a well-investigated mode of action that has important clinical implications. Many chemotherapeutic regimes are based on platinum compounds, and resistance to these drugs is a major obstacle in successful treatment of some cancers. The mechanisms that cause variation in response to therapy are not well understood, and the ability to predict sensitivity from a baseline profile of the tumor would help to improve therapy selection and thereby potentially reduce patient morbidity and mortality. We then expand our analysis to a larger set of 118 drugs to investigate whether the method is able to associate drugs with similar modes of action. We show that statistical integration conducted through a joint analysis of the data gives specific advantages in terms of sensitivity and confidence of pathway associations.

## Results

**Figure 1:**
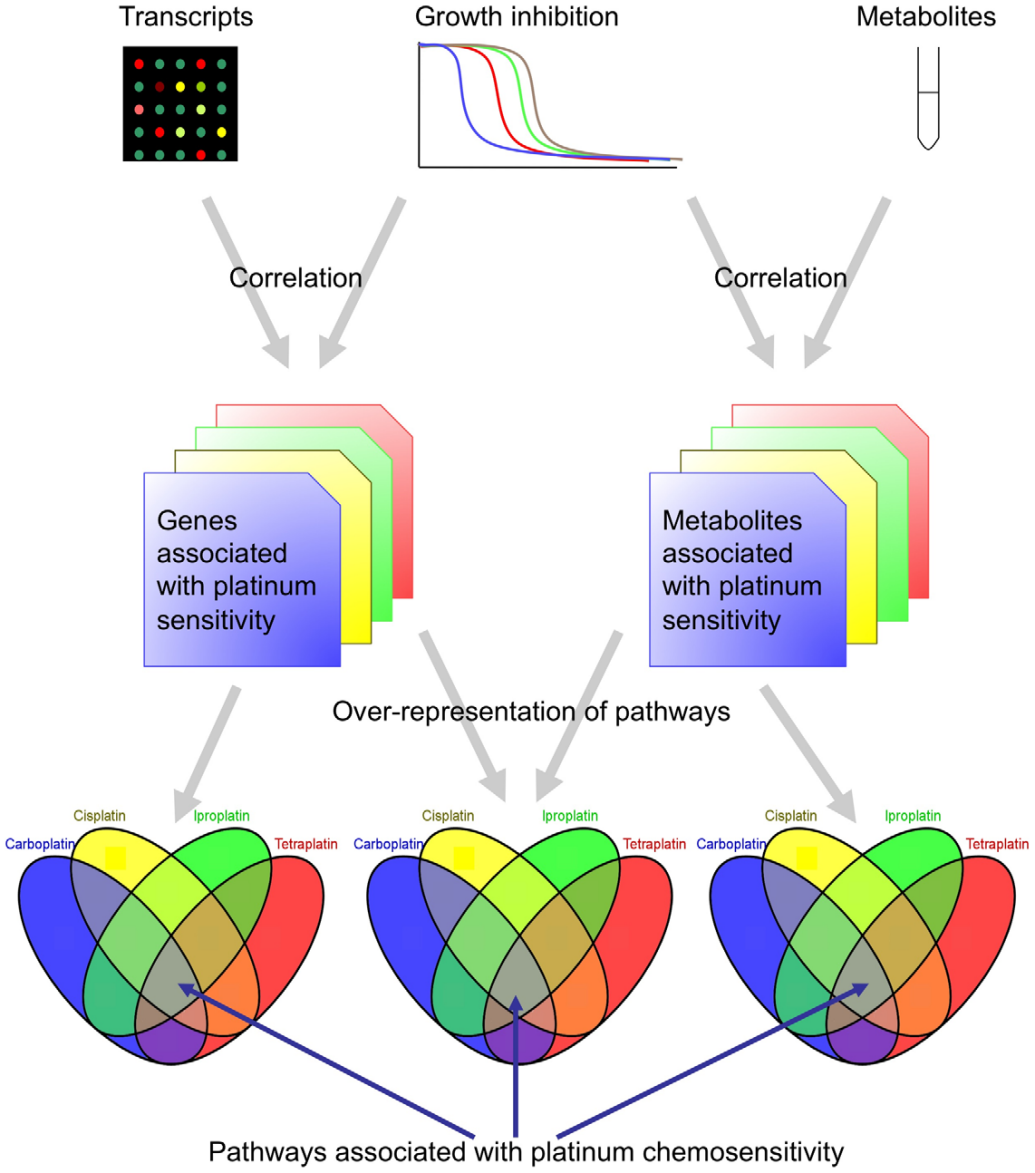
Consensus-phenotype integration of transcript and metabolite data: a schematic of the study design.

Figure 1 shows a schematic overview of our data analysis strategy. Whole genome gene expression (transcriptomic), metabolomic, and drug sensitivity data were obtained for the NCI60 tumor cell line panel. The transcriptomics data was derived using the U133 Affymetrix chip; in total 44928 probesets were measured, equating to 17150 gene products mapping to distinct UniProt identifiers, each measured across 58 cell lines [17]. The metabolomic data consisted of measurements of the total intracellular abundance of 154 uniquely identified metabolites across all 58 cell lines [18], including lipid compounds (e.g. cholesterol), glycolytic intermediates (e.g. glucose-6-phosphate), nucleic acid metabolites (e.g. adenine, uracil, hypoxanthine) and amino acids (e.g. glutamate, taurine). The full list along with our assigned KEGG IDs can be obtained in Table S4. We used drug sensitivity data (GI_50_ values indicating the concentration of the drug which inhibited cell growth by 50%) [15], [16] initially for four platinum-based chemotherapeutics, cisplatin, carboplatin, tetraplatin and iproplatin. Data for a fifth platinum drug (diaminocyclohexyl-Pt(II)) was available, and was used at a later stage as a test compound to validate our findings.

### Identifying pathways significantly associated to platinum sensitivity

For each drug we ranked all probe sets by their absolute Pearson correlation (|*r*|) to the −log(*GI_50_*) values across all cell lines. Setting the false discovery rate (FDR) [19] at 60% we then selected genes considered to be significantly associated to chemosensitivity. A high FDR was tolerated at this stage of the analysis to ensure that subsequent pathway analysis was adequately powered. Repeating this process for the metabolite data we obtained separate panels of genes and metabolites that were deemed to be associated with the sensitivity to each drug (see Table S1). In total 3, 33, 37 and 92 metabolites and 915, 1620, 5035 & 6533 genes were identified as associated with sensitivity to carboplatin, cisplain, iproplatin and tetraplatin treatment respectively.

To assess which pathways characterized the drug sensitivity phenotype we then performed OR analysis with pathways from the ConsensusPathDB [20]. The ConsensusPathDB collates pathways from several public databases of protein interactions, signaling and metabolic pathways as well as gene regulation in humans. We restricted our analysis to sources covering biochemical reactions: KEGG [21], Reactome [22], Netpath (http://www.netpath.org), Biocarta (http://www.biocarta.com), HumanCyc [23] and the pathway interaction database (PID) [24]. The use of multiple databases reduces bias by enhancing coverage. At the time of analysis the ConsensusPathDB contained 1875 pathways from the selected sources, of which 1651 contain at least one gene and 581 contain at least one metabolite measured in the NCI60 data (excluding the highly prevalent ‘currency’ metabolites phosphate, diphosphate and NADP+). OR analysis of the phenotype-associated gene panels indicated that 63, 74, 233 and 242 pathways were associated with cisplatin, carboplatin, iproplatin and tetraplatin sensitivity respectively (p<0.05). The equivalent analysis for metabolite panels indicated that 24, 13, 4, & 5 pathways were associated with these phenotypes.

### Consensus pathway and inter-omic integration

**Figure 2:**
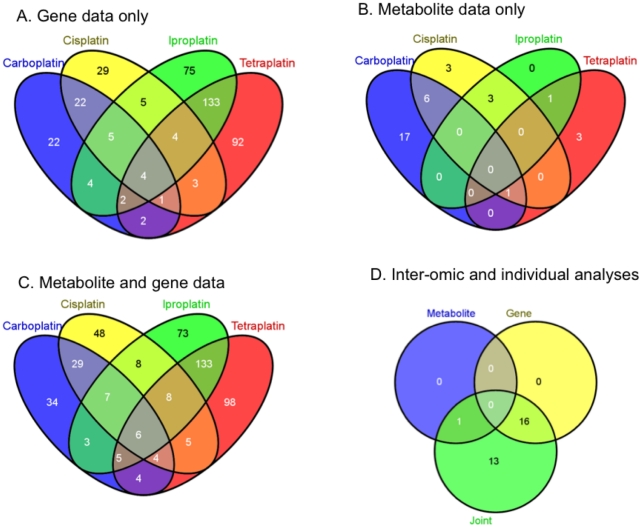
Consensus-phenotype and inter-omic integration at the pathway level. The numbers of common pathways significantly over-represented for each compound are shown as Venn diagrams. A transcript data, B metabolite data, C inter-omic analysis using both metabolite and transcript data and D comparison of the three approaches using pathways which are significant for at least three drugs. (All Venn diagrams produced by **Venny** [**55**]).

To highlight pathways relevant to general platinum sensitivity, as opposed to particular platinum compounds, we looked for pathways that were associated with more than one drug response phenotype (‘consensus-phenotype integration’; Figure 2 A & B). Within the gene transcript analysis (Figure 2A), the drugs appeared to divide into two pairs that shared many pathways in common. Iproplatin and tetraplatin were most similar, sharing 143 (133+4+4+2) of the 330 (75+5+5+4+143+92+3+1+2), ie. 43% of the pathways associated with either drug (Figure 2A). Carboplatin and cisplatin also show a high level of similarity (32 of 103 pathways, 31%). In the metabolic analysis (Figure 2B) the similarity between iproplatin and tetraplatin was much lower, while carboplatin and cisplatin retained a high level of similarity with 7 of the 30 pathways being shared (23%). The gene analysis highlights many more pathways than metabolite analysis due to both the higher number of pathways with sufficient numbers of quantified transcripts and the limited number of quantified and identified metabolites.

We next combined the transcriptomic and metabolomic data into a joint inter-omic OR analysis (Figure 2C) by estimating the joint probability of association of each pathway with the drug sensitivity phenotype assuming independence between the probability of association from the gene and metabolite data separately (see Methods). 35 pathways were found to be significant for at least one drug in the joint analysis that did not feature in either of the separate analyses of gene expression or metabolite levels. To confirm the significance of the increase in pathway detection after integration of the metabolic and transcriptomic data, we estimated the null distribution of the joint analysis probabilities by permuting the gene analysis pathway probabilities relative to the metabolite analysis pathway probabilities. For carboplatin only 3 of the 100 permutations produced more pathways than the real data and for cisplatin no permutations produced as many pathways as the real data. However, for iproplatin and tetraplatin, the number of pathways detected was not significantly enhanced by the joint OR analysis, suggesting that the combined analysis may be most advantageous when the numbers of significantly associated genes or metabolites are relatively low.

To examine the significance of the numbers of pathways in the joint OR analysis that were commonly associated to the effect of multiple drug treatments, two null models were generated. Null model I assumed that genes and metabolites identified as significantly associated to a phenotype were randomly selected whereas null model II correspondingly assumed that pathways are selected randomly. Table 1 summarizes the pathway coincidence between the output of joint OR analysis across the four platinum drugs for these two null models compared to the real data and reports the associated FDR in each analysis. We observed that by setting our criterion of significant association between a pathway and platinum sensitivity at requiring a majority of the drugs to be associated with that pathway (i.e. at least 3/4) we achieved acceptable FDRs of 0.2% (null model I) and 16.9% (null model II, the most extreme scenario).

**Table 1:** Null models I and II.

	Number of pathways common to exactly *n* drugs and FDR			
Number of drugs, *n*	1	2	3	4
**Real data (inter-omic analysis)**	251	182	24	6
**Null model I**	161.8	6.6	0.06	0.0
**Cumulative false discovery rate**	36.4%	3.2%	0.2%	0.0%
**Null model II**	539.3	81.2	5.0	0.1
**Cumulative false discovery rate**		40.7%	16.9%	<0.1%

The numbers of pathways associated with exactly *n* drugs for each of the null models and in real data.

Using the majority overlap criterion we compared the number of pathways consistently associated with platinum sensitivity between the individual and joint analyses (Figure 2D). The joint OR analysis identified all pathways highlighted by the individual –omic OR analyses combined (17 in total), but also indicated a further 13 pathways that were consistently associated (+76%). No pathways were found to be common between both the separate gene and metabolite analyses.

Overall 30 pathways met the majority criterion of association with sensitivity at least 3 platinum drugs and hence general platinum cytotoxicity (Table 2; Figure 2C & D). All the databases used to compile the ConsensusPathDB contributed pathways to the final selected consensus pathways, highlighting the value of the ConsensusPathDB strategy in pathway analysis. While this subset of pathways included those with established relationships to platinum sensitivity and general chemosensitivity, such as DNA repair and Akt regulation of nuclear transcription, there were also several pathways related to metabolic processes not previously reported as determinants of platinum sensitivity. These included nucleotide metabolism, fatty acid, triglyceride and lipid metabolism.

**Table 2:** Pathways significant by over representation analysis with respect to platinum drug sensitivity.

	Effective size of pathway in terms of…		Number of drugs with this pathway over-represented in…		
Pathway and *source database*	genes	metabolites	Inter-omic analysis	gene analysis	metabolite analysis
**Metabolic Pathways**					
	**22**	**2**	**3**	**2**	**0**
**Triacylglyceride Biosynthesis ** ***Reactome***	**29**	**3**	**4**	**3**	**0**
**Purine metabolism ** ***Reactome***	**82**	**16**	**3**	**2**	**1**
**Purine metabolism - Homo sapiens (human) ** ***KEGG***	**412**	**19**	**3**	**2**	**1**
**DNA Repair ** ***Reactome***	**144**	**5**	**3**	**1**	**1**
**Hormone-sensitive lipase (HSL)-mediated triacylglycerol hydrolysis ** ***Reactome***	**23**	**2**	**3**	**1**	**2**
**Lipid and lipoprotein metabolism ** ***Reactome***	**243**	**9**	**3**	**2**	**0**
**De novo biosynthesis of pyrimidine deoxyribonucleotides ** ***HumanCyc***	**17**	**1**	**3**	**1**	**0**
**Salvage pathways of purine and pyrimidine nucleotides ** ***HumanCyc***	**45**	**9**	**3**	**1**	**0**
**Phosphatidylcholine biosynthesis pathway ** ***BioCarta***	**6**	**1**	**3**	**2**	**0**
Base Excision Repair *Reactome*	27	3	4	2	2
Nucleotide metabolism *Reactome*	137	27	3	2	1
Pyruvate metabolism and TCA cycle *Reactome*	52	6	3	3	0
Reversible phosphorolysis of pyrimidine nucleosides *Reactome*	2	3	3	0	3
Phospholipid biosynthesis II *HumanCyc*	57	4	3	3	0
**Non-Metabolic pathways**					
**Signaling in Immune system ** ***Reactome***	**467**	**1**	**3**	**2**	**0**
**Hemostasis ** ***Reactome***	**390**	**1**	**3**	**2**	**0**
Rho GTPase cycle *Reactome*	265	0	4	4	N/A
lck and fyn tyrosine kinases in initiation of tcr activation *BioCarta*	18	0	3	3	N/A
AKT phosphorylates targets in the nucleus *Reactome*	31	0	3	3	N/A
TCR signaling in naïve CD8+ T cells *PID*	107	0	3	3	N/A
TCR *NetPath*	267	0	4	4	N/A
BCR *NetPath*	316	0	3	3	N/A
Immunoregulatory interactions between a Lymphoid and a non-Lymphoid cell *Reactome*	138	0	3	3	N/A
amb2 Integrin signaling *PID*	98	0	3	3	N/A
Apoptotic dna-fragmentation and tissue homeostasis *BioCarta*	17	0	4	4	N/A
Apoptotic cleavage of cell adhesion proteins *Reactome*	16	0	3	3	N/A
Notch receptor binds with a ligand *Reactome*	20	0	3	3	N/A
Receptor-ligand binding initiates the second proteolytic cleavage of Notch receptor *Reactome*	22	0	3	3	N/A
Integrin cell surface interactions *Reactome*	171	0	4	4	N.A

Pathways shown are those significantly over-represented in at least 3 drug lists for the inter-omic analysis. N/A indicates no quantified metabolites were present in the pathway. Rows shown in bold are those where the inter-omic analysis means that the pathway is significantly associated with chemosensitivity to more drugs than the individual analyses combined. The pathways are split into two classes, metabolic and non-metabolic and then ordered so that the pathways with improved detection in the inter-omic analysis are at the top of the table.

**Figure 3:**
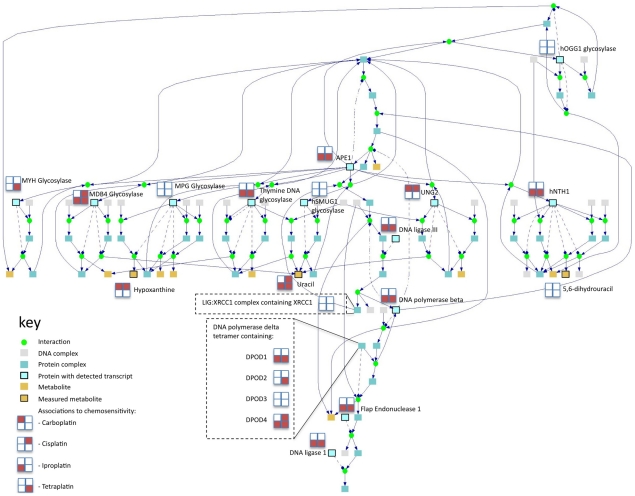
Base Excision Repair Pathway (Reactome). The Pathway diagram was generated using ConsensusPathDB [20]. All quantified metabolites and transcripts are marked and the drugs with which they appeared associated are shown. A solid line indicates a substrate or product (or protein participating in a protein complex) and a dotted line shows an enzymatic link.

The added value of the inter-omic OR analysis prior to consensus phenotype integration can be more clearly discerned at the individual pathway level. Figure 3 is a network representation of the base excision repair (BER) pathway from Reactome and depicts both the detected entities and the drugs with which each detected entity is associated. While the majority of entities were significantly associated to the effect of at least one of the four platinum agents, there was significant variation in the pattern of association and no gene or metabolite was significantly associated to all four treatments. Accordingly the pathway was only significantly associated to tetraplatin and iproplatin sensitivity using the transcriptome data alone, and to carboplatin and cisplatin sensitivity using the metabolite data in isolation. Using the joint OR analysis the BER pathway was significantly associated to the sensitivity to all four platinum compounds (Table 2) and the evidence for association with each drug was increased, due to the added information from the alternative data type. Of the 12 pathways for which inter-omic OR analysis improved the consensus between the drugs, 10 refer to metabolic processes.

In order to validate and to test the generalisability of our findings we then examined GI_50_ data from a test compound, diaminocyclohexyl-Pt(II). After conducting the same inter-omic OR analysis as described previously, we observed that the effects of this compound on the NCI60 panel was associated with 5 of the 6 pathways common to all 4 other platinum drugs along with a further 12 pathways from Table 2 and 90% (220/245) of the pathways associated with diaminocyclohexyl-Pt(II) were Associated with at least one of the other platinum drugs. In particular there were 138/152 pathways commonly associated between diaminocyclohexyl-Pt(II), iproplatin and tetraplatin sensitivity. Since OR analysis makes no distinction between positive and negative molecule/sensitivity correlations, we also examined the direction of associations between the metabolites detected in the consensus pathways and the GI_50_ of all platinum drugs (Table 3). In total, a panel of 22 metabolites were associated with the consensus metabolic pathways from analysis of the four training compounds. While there was variation in the metabolites associated with specific treatments, where a significant association was observed the direction of correlation was consistent across the training set. The GI_50_ values of our test compound, diaminocyclohexyl-Pt(II), was significantly correlated to 19/22 metabolites in this panel, with complete consistency in the direction of association with the training set data.

**Table 3:** Metabolites involved in the pathways from Table 2, showing the direction of association (if above the FDR cutoff) and r, the correlation coefficient to the −log(GI50) values.

	Carboplatin	Cisplatin	Iproplatin	Tetraplatin	Diaminocyclohexyl-Pt II
2-oxoglutarate	0.18	**↑** 0.26	0.20	0.12	**↑** 0.16
Adenine	0.01	−0.01	0.13	**↑** 0.21	**↑** 0.15
β-alanine	−0.13	−0.04	0.05	**↑** 0.28	**↑** 0.23
Citrate	0.00	0.01	0.23	**↑** 0.21	**↑** 0.19
CMP	−0.04	−0.04	0.15	**↑** 0.22	**↑** 0.21
dUTP	0.20	**↑** 0.25	0.20	**↑** 0.24	**↑** 0.25
L-glutamate	0.00	0.09	0.02	**↑** 0.23	**↑** 0.22
Phosphoenol-pyruvate	0.00	0.09	**↑** 0.26	**↑** 0.33	**↑** 0.30
S-adenosyl-L-methionine	0.02	−0.01	0.20	**↑** 0.27	**↑** 0.20
Taurine	−0.14	−0.03	0.11	**↑** 0.45	**↑** 0.35
Cholesterol	−0.16	**↓** −0.25	**↓** −0.35	**↓** −0.37	**↓** −0.38
Deoxyuridine	0.03	0.02	−0.02	**↓** −0.21	**↓** −0.18
Glycerol	−0.21	**↓** −0.27	**↓** −0.43	**↓** −0.38	**↓** −0.36
Guanine	−0.02	−0.04	−0.15	**↓** −0.20	**↓** −0.18
Guanosine	−0.06	−0.16	**↓** −0.30	**↓** −0.24	**↓** −0.30
Hexadecanoic Acid	0.03	−0.09	**↓** −0.26	**↓** −0.22	**↓** −0.27
Hypoxanthine	**↓** −0.36	**↓** −0.36	−0.13	−0.05	−0.04
Inosine	−0.21	**↓** −0.25	−0.07	−0.05	−0.10
Uracil	−0.19	**↓** −0.25	**↓** −0.28	**↓** −0.19	**↓** −0.21
Urea	−0.14	**↓** −0.25	**↓** −0.31	**↓** −0.36	**↓** −0.37
Uridine	**↓** −0.38	**↓** −0.34	−0.17	**↓** −0.18	**↓** −0.20
Xanthine	**↓** −0.32	**↓** −0.25	−0.13	−0.07	−0.02

### Global analysis of chemosensitivity pathways

**Figure 4:**
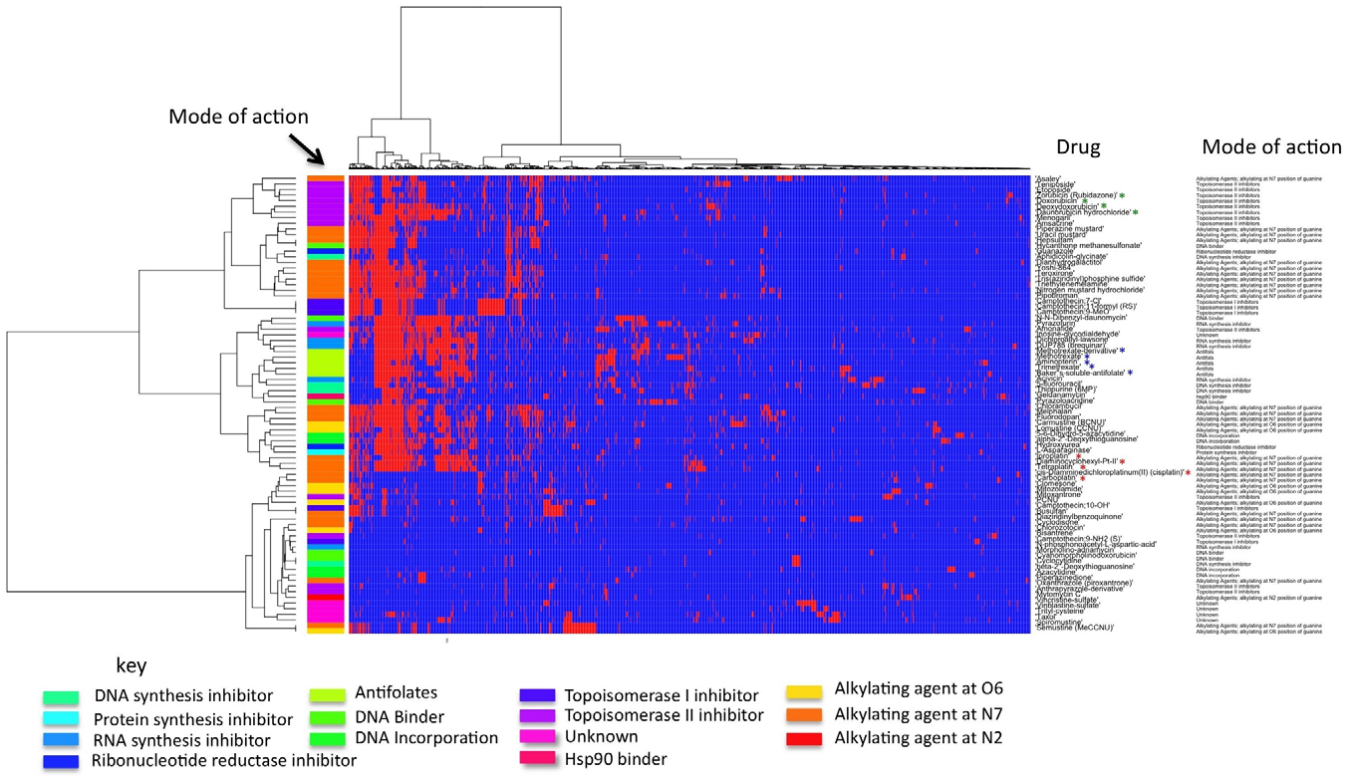
Clustering drugs according to the pathways significantly correlated to sensitivity. Red and blue indicates a pathway which is or is not significant in the inter-omic analysis. A significance level of p<0.05 was used for each pathway. Clustering was performed using complete linkage and the Hamming distance metric. Pathways not associated with any drug have been omitted from the figure. Red asterisks indicate platinum drugs, blue asterisks indicate antifolates and green asterisks indicate anthracycline-based drugs.

To explore more broadly the relationships between chemosensitivity and biological pathways across a range of agents, and to ascertain the specificity of the consensus phenotype analysis for platinum sensitivity pathways, inter-omic OR analysis was performed using GI_50_ data for all 118 compounds available within the NCI 60 dataset. In total 1262 pathways were significantly associated with the drug sensitivity of at least one compound, while 82 compounds gave at least one significant pathway. Figure 4 shows the clustered heat-map of the binary association matrix in which each element is set to one if a pathway is significantly associated with sensitivity to a given drug and zero otherwise (see Table S2). Significant clustering of the drugs according to mode of action is visible. For example the dihhryofolate reductase inhibitor methotrexate co-clustered with related compounds aminopterin, trimetrexate, and Baker's-soluble-antifolate (triazinate) (Figure 4, blue asterisks); the sensitivities to all four compounds were associated with 91 common pathways. While one might expect structural analogues such as these to produce a similar pattern of sensitivity and hence similar pathway associations, structurally unrelated compounds that share a common molecular target also co-clustered in certain instances. One interesting observation was the similarity in pathway association, reflected by common membership of a cluster, of several structural analogues, the anthracycline-based compounds, (doxorubicin, zorubicin, danorubicin hydrochloride and deoxydoxorubicin) with the podophyllotoxin-based etoposide and teniposide (Figure 4, green asterisks). The four anthracyclines in the cluster share a large proportion of associated pathways: 63 of the 401 pathways associated with any of the anthracyclines are commonly associated to the effect of all four compounds, and of these, 60 are also associated to the chemosensitivity to either teniposide or etoposide. Etoposide and its derivatives directly inhibit topoisomerase II activity, followed by induction of DNA strand breaks and selective cytotoxicity in tumour cells [25] whereas anthracyclines intercalate DNA, indirectly inhibiting the progression of topoisomerase II and blocking replication [26]. Thus, the inter-omic pathway analysis is apparently able to associate chemosensitivity phenotypes on the basis of a common pathophysiological link independent of whether the key molecular targets are affected directly, or indirectly by an upstream process.

While such mechanistic relationships were readily observable, the most prominent division between the compounds, visible as the two largest clusters in Figure 4, appeared to be separating on the overall frequency of pathways associated with chemosensitivity, with the top cluster in the diagram possessing on average 2.95 times the number of positive associations of the lower cluster. While each of the five platinum compounds in the dataset were most similar in pathway associations to another platinum compound, they were separated across the two largest clusters with cisplatin and carboplatin forming one group and tetraplatin, iroplatin and diaminocyclohexyl-Pt II another. This separation agreed with the low numbers of common chemosensitivity pathways between members of these two groups in earlier analyses (Figure 2). Thus, the clustering structure did not describe associations common across the platinum compounds, illustrating the difficulty of using clustering approaches alone to identify pathways that may determine class-specific chemosensitivity and the advantages of the consensus phenotype approach.

To assess the specificity of the identified consensus platinum-sensitivity pathways we compared these to the most frequently associated pathways in the global inter-omic OR analysis (Table S2). Of the 54 (top 50 including ties) most frequently associated pathways (Table S3), just seven intersect with pathways identified by consensus phenotype integration, mostly related to immunoregulatory processes (“T-cell receptor” – Netpath; “B-Cell receptor” – Netpath; “Rho GTPase cycle” – Reactome; “lCK and FYN tyrosine kinases in initiation of TCR activation” – BioCarta; “AMB2 integrin signalling” – PID; “Immunoregulatory interactions between a Lymphoid and a non-Lymphoid cell” – Reactome; and “TCR signalling in naive CD8 T cells” – PID). Hence the remaining 23/30 consensus platinum-sensitivity pathways, dominated by metabolic processes, are not associated with sensitivity to a wide range of chemotherapeutic agents and are more likely to be specific to platinum sensitivity.

## Discussion

Our results show that an inter-omic, consensus phenotype approach to integration of molecular profiles can reveal a cellular metabolic phenotype robustly associated with platinum chemosensitivity across the NCI-60 cell line panel. Many of the specific aspects of this phenotype are consistent with the perturbations described across many studies of tumour cell metabolism, and several of these have been associated with the development, or likely acquisition, of drug resistance phenotypes. The classic hallmark of tumour cell metabolism is the Warburg effect: an increase in glucose uptake and glycolysis to lactate even in normal oxygen conditions. In addition to the Warburg effect tumour cells are frequently reported as exhibiting higher rates of glutaminolysis, fatty acid and lipid metabolism, and nucleotide synthesis [27]. Our observations from the NCI-60 molecular profiles suggest a positive correlation between all of these phenotypes and platinum chemosensitivity.

**Figure 5:**
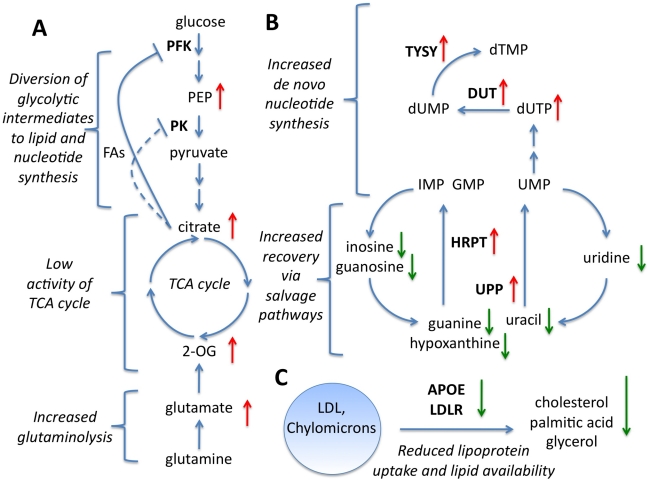
Processes associated with platinum sensitivity. Three processes associated with platinum sensitivity, the arrows indicate the direction of correlation to the −log(GI50) values for that gene or metabolite. **A** Energy metabolism. **B** Nucleotide *de novo* synthesis and salvage. **C** Lipid uptake.

Figure 5 summarises some of the key correlations observed between gene transcription, metabolite levels and platinum sensitivity from the consensus pathways indicated by our analysis. The relatively higher levels of citrate and phosphoenolpyruvate (PEP), observed in more sensitive cell lines (Figure 5A), are consistent with low TCA cycle activity (via product inhibition) and increased diversion of glycolytic intermediates into anabolic pathways such as the pentose phosphate which feeds nucleotide synthesis [28]. Under these conditions tumour cells increase the uptake of glutamine and its conversion to oxaloacetate via glutamate and 2-oxoglutarate (2-OG) in order to replace TCA cycle intermediates and NADPH [29]. Both glutamate and 2-OG levels were also higher in more sensitive cell lines. Thus more ‘Warburg–like’ cells appear more sensitive to platinum treatment than less metabolically transformed lines. The selection of TCA cycle and pyruvate metabolism as a sensitivity pathway in our analysis is likely to reflect these associations.

The dependency of tumour cells on glycolysis for synthetic intermediates could be exploited in platinum chemotherapy; for example the clinically-relevant glycolysis inhibitor 2-deoxy-glucose (2-DG) has been shown to enhance cisplatin cytotoxicity in head and neck cancer cells [30]. Interestingly, this synergy appeared to be mediated in part via oxidative stress, a process that would lead to DNA lesions (e.g. 8-oxo-2′-deoxyguanosine) requiring base excision repair (BER) which was one of the key consensus sensitivity pathways selected by our analysis (Figure 3). While it is clear that nucleotide excision repair (NER) capacity is linked to cisplatin resistance [31], [32], [33]; it is becoming evident that BER is also important in the effect of cisplatin derived drugs [34]. Cross-linking of DNA via platinum derived drugs can increase the production of free radicals by disrupting the cellular redox balance [35]. We suggest that the association of the BER pathway with four platinum drugs observed in the present study is related to increased ROS production and not adduct formation (repaired by NER). Intracellular levels of ROS seem vital to the cytotoxic effect of the platinum derived drugs, further evidenced by the fact that oxaliplatin (a later generation of Pt drug) is highly cytotoxic but forms less platinum-DNA adducts compared to equal amounts of cisplatin [35].

A particularly high degree of coordination between gene transcript and metabolite levels was observed in nucleotide metabolism, revealing a robust association between increased nucleotide synthesis, both *de novo* and via recovery of catabolic intermediates, and tumour cell Pt sensitivity (Figure 5B). For example, in the *de novo* pathway, we observed a positive correlation between levels of dUTP (a precursor to dTMP), expression of dUTP pyrophosphatase (DUT r = 0.38), expression of thymidylate synthase (TYSY r = 0.27) and platinum sensitivity. dUTP has to be hydrolysed to dUMP by DUT to prevent the incorporation of uracils into DNA and suppression of DUT has been shown to sensitize cells to other chemotherapeutics such as pyrimidine anti-metabolites [36].

Increased expression of nucleotide salvage pathway enzymes (e.g. uracil phosphoribosyl transferase or UPP (r = 0.20), hypoxanthine-guanine phosphoribosyl transferase or HPRT, r = 0.27) in sensitive cell lines was accompanied by decreases in several intermediates of purine and pyrimidine catabolism (namely guanine, guanosine, hypoxanthine, inosine, uracil, uridine and urea) and increase in CMP, the nucleotide product of HPRT. Kowalski et al. [37] have shown clear links between inactivation of salvage pathway enzymes such as HGPRT or loss of feedback inhibition to AMP and GMP *de novo* synthesis and cisplatin resistance in yeast. Interestingly in the same study the addition of low concentrations of extracellular purines also abolished cisplatin cytotoxicity; thus the metabolome may have a causal influence on platinum sensitivity and not just represent epiphenomena that is a passive consequence of aberrant cell division.

Our pathway analysis also predicts that lipid metabolism has a direct impact on chemosensitivity. We observed lower cholesterol, glycerol, and hexadecanoic acid (palmitate) in more sensitive cell lines, together with negative correlations between expression of apolipoprotein E (APOE; mean R = −0.21), LDL receptor (LDLR; mean R = −0.27) and platinum sensitivity (Figure 5C). All these observations are consistent with a hypothesis that increased uptake of lipoproteins and constituent triglycerides, fatty acids and cholesterols can confer resistance to platinum, a phenomenon previously shown in drug resistant leukemic cell lines [38]. A related pathway highlighted as associated with sensitivity was phosphatidylcholine biosynthesis. We observed a positive correlation between choline kinase (CK, r = −0.28, correlation to −log(GI50)) expression and resistance to platinum. Recent work by Shah et al. [39] in breast cancer cells have shown that CK regulates pro-survival MAPk and PI3K/Akt signaling via phosphatidic acid, and that overexpression leads to drug resistance.

While previous pathway analysis was conducted on gene expression profiles alone from the NCI60 dataset [40], the use of correlation analysis and the combination of metabolite and gene transcription measures in our study provides an unprecedented level of detail into the contribution of metabolic pathways to drug sensitivity. Using gene set enrichment analysis (GSEA), Reidel et al. [40] suggested that, in addition to a number of cell signaling and survival networks, methionine metabolism may contribute to chemotherapeutic resistance to multiple agents, while fatty acid and β-alanine metabolism were specifically associated with platinum-resistance. In the context of fatty acid metabolism we show here that lipid uptake and processing may in fact be the driving factor in this association. It is also interesting to note that although we did not observe over-representation of β-alanine and methionine metabolic pathways, both β-alanine and S-adenosylmethionine levels were significantly positively correlated to platinum sensitivity, adding functional evidence in support of these earlier findings.

At present, our study is one of very few that presents a strategy for simultaneous interpretation of gene expression data, metabolic profiles and physiological endpoints using biological pathway analysis, and has several advantages over other approaches. Multivariate analysis using pattern recognition algorithms such as PCA, [41], PLS [42] and Kohonen Networks (Self-Organising maps) [43], have been shown to be useful in revealing novel associations between “-omics” datasets, but fail to take into account prior biological knowledge relevant to the phenomenon at hand - a feature which is clearly present in pathway-based techniques. Gene and metabolite coregulation at the pathway level has been previously studied using OR analysis [44], [45]. Transcripts significantly correlated to metabolite levels were examined for over-representation of Gene Ontology terms [46] or pathways (defined by MapMan BINS). Bradley and Gibons' work reveals a degree of coordination present between transcriptional and metabolic measurements at a pathway level, a necessary prerequisite for our approach to be successful. Importantly none of these examples use a function physiological endpoint (cytotoxicity) as driver in pathway selection, leading to a consensus phenotype description of the phenomenon of interest. We show here that such an approach is critical in reducing false positive selection of pathways.

All OR techniques share the limitation that they rely on a database containing pre-defined pathways, and therefore cannot identify novel pathways or functional modules. In this work we have tried to overcome this limitation somewhat through deconstructing the pathways which were significantly associated and then functionally interpreting the elements of the pathways which showed significant associations (Figure 5). However, even this requires that the elements of the process are sufficiently grouped in existing pathways to allow for those pathways to be significantly associated.

Ultimately, a systems biology approach, such as the inter-omic pathway analysis presented in our study, could assist the development of anti-resistance chemotherapeutic strategies, and better individualization of treatment, i.e. personalized medicine. Using gene expression models (GEMs) based on cytotoxicity in the NCI-60 panel, Williams et al. [47] were able to stratify tumour response and/or patient survival in seven independent cohorts of patients with breast, bladder and ovarian cancer. Crucially, the *in vitro* derived GEMs outperformed those derived directly from *in vivo* data. Recently it has also been shown that pre-treatment metabolic profiles can be used to predict the metabolic fate or effect of drugs in rodents [48], healthy humans [49], [50] and breast cancer patients [51]. Given that the metabolic phenotype of cancer is already the basis of imaging techniques such as FDG-PET that are currently used to detect early responses to therapy, there is potentially great value in combing such pharmaco-metabonomic studies with other characterization of the patient or tumour genome and it is our belief that the integration of molecular profile data yields more than the sum of its parts. It remains to be seen if the combination of “-omics” data provides a competitive advantage over targeted biomarker studies for prognosis and prediction of drug response in oncology. It remains to be seen if the combination of “-omics” data provides a competitive advantage over targeted biomarker studies for prognosis and prediction of drug response in oncology. Several major challenges to such approaches and translation from in vitro studies, in particular tumour heterogeneity, require further study. However, irrespective of biomarker development, the knowledge that chemotherapeutic sensitivity is in part determined by the metabolic phenotype suggests that metabolic enzymes may be potential targets in oncology for both drug naive and chemoresistant patients.

## Methods

### NCI60 and pathway data

The NCI60 data was downloaded from http://dtp.nci.nih.gov/mtargets/download.html on 27th August 2008. For this work three datasets were used: metabolite levels, gene expression levels and drug sensitivities. The metabolite data consists of measurements of 352 metabolites, 154 identified, across 58 cell lines, performed by Metabolon Inc. [52]. The transcriptomics data was obtained using the U133 Affymetrix chip by Genelogic [17]. 44928 probesets were measured, equating to 17150 genes mapping to distinct UniProt identifiers, measured across the same 58 cell lines. The drug resistance data was selected from the 118 ‘mechanism of action’ drugs data [15], [16]. Each compound was profiled in between 2 and 1176 independent experiments in a 48-hour sulforhodamine B assay. The values used are the −log(GI_50_), where GI_50_ is the dosage of the drug which inhibits the growth of the cells by 50%. . GI_50_ values were averaged across the replicates for each cell line, thus increasing the robustness of the primary phenotypic endpoint.

Pathways were derived from the ConsensusPathDB [20] which assimilates pathways from a range of public databases (see Results). Gene IDs are mapped to UniProt [53] protein IDs. For metabolites, where available KEGG [21] compound IDs were used, else, ChEBI [54] IDs were used.

### Construction of gene/metabolite lists and over-representation analysis

Pearson correlations were calculated between all transcript/metabolite levels and −log(GI_50_) values for each drug. Transcripts/metabolites significant below a false discovery rate threshold of 60% were retained in each test set for OR analysis. Each UniProt identifier in the ConsensusPathDB pathways can be mapped to zero or more gene identifiers on the U133 chip. The background estimate (m in equation 1) for OR analysis was adjusted to reflect the following: 1) Where ConsensusPathDB proteins could not be mapped to any gene identifiers, these were ignored; and 2) where ConsensusPathDB proteins mapped to multiple probesets measuring genes in the transcript data, the number of probesets was used. In addition, several metabolites, often referred to as “currency metabolites”, which appear in many pathways and do not provide specificity were removed before analysis. The currency metabolites removed were phosphate, diphosphate and NADP+. Thus, given the transcriptomic and metabolomic data, an “effective size”, *N_i_*, could be defined for each pathway, *I*, in terms of genes and metabolites, The effective pathway size may be larger or smaller than the actual number of proteins/metabolites in the pathway. Pathway significance was calculated using the hypergeometric distribution,
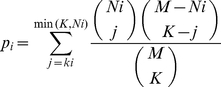
(1)where K is the number of genes or metabolites associated with the drug and k_i_ is the number of genes or metabolites from the pathway. P<0.05 was used as the criterion defining significance of pathway enrichment.

### Joint transcript and metabolite analysis

We used the pathway p-values *p_i_* from the individual analyses to combine the data. If there were no transcripts or no metabolites measured for pathway *i*, we set *p_i_* = 1 for that data type. Since the transcript/metabolite data were generated from separate experiments. We thus assumed independence of the pathway associations from the different data sets. We thus computed the joint probability *p_Ji_* of association of pathway *i* with the drug sensitivity phenotype as *p_Ji_* = *p_Gi_ p_Mi_* where *p_Gi_* and *p_Mi_* denote the probability of association from the individual gene and metabolite data separately.

### Null models

Null model 1 was generated by creating random gene and metabolite lists of matching size to those observed for each of the drugs. Standard OR analysis was then performed and then numbers of overlapping pathways were recorded. 100 sets of random lists were generated and the mean number of pathways common to different numbers of drugs were recorded in table 1.

Null model II assumes that the pathways are selected at random, and so taking the numbers of pathways selected for each drug, the exact probability of a pathway being selected for *n* drugs was calculated. To do this we calculated the probability of a pathway being selected at random from the full list of pathways, given the number of pathways selected. By calculating this for each of the drugs we have.

Additionally, we examined the added information given by the joint analysis. For each drug the lists of p-values from the metabolite and transcript analyses were randomly permuted 100 times before combination (randomizing the pathway association between the two sets). The number of times in which more pathways were significant (p<0.05) for the permuted lists than in the real data was recorded.

Cumulative false discovery rates for all models were calculated by dividing the ‘expected’ number of pathways as given by the null model, by the actual (cumulative) number of pathways found in the real data in at least *n* drugs.

## Supporting Information

Table S1 Panels of genes and metabolites that were deemed to be associated with the sensitivity to each drug.
(0.57 MB XLS)

Table S2 The binary table used to produce the heatmap in Figure 4.
(0.39 MB XLSX)

Table S3 The pathways most frequently associated with drugs from the panel.
(0.01 MB XLSX)

Table S4 List of metabolites measured in the NCI60 panel, along with our assigned KEGG IDs.
(0.05 MB XLSX)
